# Liver Histopathological Analysis of 24 Postmortem Findings of Patients With COVID-19 in China

**DOI:** 10.3389/fmed.2021.749318

**Published:** 2021-10-11

**Authors:** Huikuan Chu, Li Peng, Lilin Hu, Yixin Zhu, Jinfang Zhao, Hua Su, Lin Yao, Qingjing Zhu, Xiu Nie, Ling Yang, Xiaohua Hou

**Affiliations:** ^1^Division of Gastroenterology, Union Hospital, Tongji Medical College, Huazhong University of Science and Technology, Wuhan, China; ^2^Department of Pathology, Union Hospital, Tongji Medical College, Huazhong University of Science and Technology, Wuhan, China; ^3^Department of Medicine, University of California San Diego, La Jolla, CA, United States; ^4^Center for Life Sciences, Tsinghua University, Beijing, China; ^5^Department of Nephrology, Union Hospital, Tongji Medical College, Huazhong University of Science and Technology, Wuhan, China; ^6^Liver and Infectious Diseases Department, Wuhan Jinyintan Hospital, Wuhan, China

**Keywords:** liver histopathological analysis, COVID-19 patients, transmission electron microscopy, edematous of mitochondria, expansions of endoplasmic reticulum

## Abstract

Although the pathologic investigation of liver injury was observed in a couple of cases in China, the detailed description of liver histopathologic and ultrastructural changes in a relatively larger series of liver tissues from COVID-19 patients is lacking. Samples from the liver were obtained from 24 COVID-19 cases from February 1 to April 1, 2020. Light microscopy showed that all liver sections had different degrees of liver injury manifested as swelling of the hepatocytes, hepatocellular necrosis, steatosis, lobular inflammation, portal inflammation, dilatation of sinusoids, and so on. SARS-CoV-2 induced liver injury might be independent of pre-existing Schistosoma infection or obstructive cholestasis. Patients combined with respiratory failure had more severe hepatocellular necrosis and male patients were more susceptible to liver injury. Although coronavirus particles or viral inclusions were not detected in the liver tissues for all cases, vacuolar degenerations in hepatocytes, edematous of mitochondria with the disruption of cristae, and expansions of the endoplasmic reticulum were observed. In conclusion, pathologic changes of liver tissues provide us a further understanding of liver injury in COVID-19 patients. Changes in the liver seem to be related to the underlying diseases/conditions.

## Introduction

The Corona Virus Disease 2019 (COVID-19), which is caused by severe acute respiratory syndrome coronavirus 2 (SARS-Cov-2), has been considered as a public health emergency of international concern by the World Health Organization (WHO). Currently, SARS-CoV-2 has spread to over 200 countries and areas with 21, 294, 845 confirmed cases, including 761, 779 deaths globally until August 16, 2020 (1). COVID-19 mainly affects the lower respiratory tract. Besides, it also induces acute kidney injury and liver dysfunction in some patients (2–5).

On the basis of previous reports from China, a subset of COVID-19 patients had liver impairment with abnormal levels of alanine aminotransferase (ALT) and/or aspartate aminotransferase (AST) accompanied by slightly elevated bilirubin levels during disease progression (6–8). The incidence of liver injury in COVID-19 patients was common, especially in some severe and deceased cases (8–10). The pathologic investigation of liver injury was observed in several studies worldwide (11–15). They centered on the pathologic findings using light microscopy and only one paper described the change of organelles using electron microscopy (15). Besides, pathologic investigation of liver injury was observed only in a couple of cases in China (16), the detailed description of pathologic findings using both light microscopy and transmission electron microscopy observations in a relatively larger series of liver tissues from COVID-19 patients in China is lacking. In this study, we report on our experience of liver findings at autopsy in 24 deceased patients with COVID-19.

## Methods

### Patients

This study was conducted in accordance with the principles of the Declaration of Helsinki and approved by the Institutional Ethics Board of Union Hospital, Tongji Medical College, Huazhong University of Science and Technology (2020. 0043-1). Written informed consent was obtained from the next of kin of each enrolled case. According to the World Health Organization Interim Guidance, the diagnosis of COVID-19 was confirmed based on real-time reverse-transcriptase polymerase-chain-reaction (RT-PCR) assay for nasal and pharyngeal swab specimens (9). Their electronic medical records, the results of the laboratory, and imaging examinations were all obtained and reviewed. The degree of severity of liver pathological changes was defined using pathologic diagnosis and the grading guideline of common pathological changes after liver transplantation (II) (17) and Scheure scoring system (18, 19).

### Tissue Sampling and Processing

Samples from the liver were obtained with ultrasound guidance and percutaneous multiple punctures of the liver from 24 COVID-19 cases with a postmortem interval of 2 h from February 1 to April 1, 2020. Tissue specimens were fixed in 3.7% formaldehyde for 48–72 h before the following procedures. Four micrometer sections were cut and stained with hematoxylin-eosin. Slides were examined and analyzed by two pathologists blinded to the study.

For EM examination, tissue specimens were fixed in 2.5% glutaraldehyde for 24–48 h. After 1% osmic acid post-fixation and gradient dehydration, Epon-embedded, toluidine blue-stained “semi-thin” sections were examined, and selected areas were chosen for thin sections. Thin sections were then cut and stained with uranyl acetate and lead citrate. EM grids were then viewed with a transmission electron microscope (HT-7800; Hitachi, Tokyo, Japan).

### Real-Time Reverse Transcription Polymerase Chain Reaction Assay for SARS-COV-2 in Tissue

Formalin-fixed, paraffin-embedded (FFPE) tissue blocks were used for RNA extraction. Total RNA was extracted using a sample RNA isolation kit (Catalog No. ADx-FF04, fromIde Biomedical Technology Co., Ltd, Xiamen, China) and a real-time reverse transcriptase polymerase chain reaction (real-time RT-PCR) assay was run on the Mx3000P qPCR system with a 2019-nCoV nucleic acid detection kit (Catalog Z-RR-0479-02-25, from Shanghai Zhijiang Biotechnology Co., Ltd, Shanghai, China) according to the manufacturer's protocol.

Two target genes, the RdRp, nucleocapsid protein (N) and E genes, were simultaneously amplified and monitored during the real-time RT-PCR assay. The primers for target 1 (RdRp) were RdRp_SARSr-F 5′-GTGARATGGTCATGTGTGGCGG-3′, RdRp_SARSr-R 5′-CARATGTTAAASACACTATTAGCATA-3′, RdRP_SARSr-P1 5′-CCAGGTGGWACRTCATCMGGTGATGC-3′, RdRp_SARSr-P2 5′-CAGGTGGAACCTCATCAGGAGATGC-3′ and E_Sarbeco_F 5′-ACAGGTACGTTAATAGTTAATAGCGT-3′. The primers for target 2 (N) were N_Sarbeco_R 5′-GAGGAACGAGAAGAGGCTTG-3′ and N_Sarbeco_P 5′-ACTTCCTCAAGGAACAACATTGCCA-3′ and the primers for target 3 (E) were N_Sarbeco_F 5′-CACATTGGCACCCGCAATC-3′, E_Sarbeco_R 5′-ATATTGCAGCAGTACGCACACA-3′ and E_Sarbeco_P1 5′-ACACTAGCCATCCTTACTGCGCTTCG-3′. A cycle threshold (Ct) value of 43 or less was defined as a positive, and a Ct value of more than 43 was defined as a negative. These testing criteria were based on recommendations by the National Institute for Viral Disease Control and Prevention (China) (http://ivdc. chinacdc.cn/kyjz/202001/t20200121_211337. html). Positive and negative controls were included.

### Statistical Analysis

Categorical variables were presented as numbers and percentages. Continuous variables were expressed as median (25th−75th percentile). The relationships of pathologic changes and clinical liver function and inflammatory data were examined using Spearman Rho's (ρ) correlation. Pathologic features among those with and without macrovesicular steatosis/liver fibrosis and among those with and without antiviral intervention/combination therapeutic intervention/combined diseases were compared using Fisher's exact test. Pathologic features among those with different degrees of liver injury and among those with and without antiviral intervention/combination therapeutic intervention/combined diseases were compared using the Mann–Whitney U test. All statistical analyses were performed using SPSS (Statistical Package for the Social Sciences) (version 25.0, IBM Corp, Armonk, NY, USA). A significance level of *P* ≤ 0.05 was used for all models (two-sided).

## Results

### Clinical Information of the Patients

The 24 patients with COVID-19 included 16(67%) males and 8(33%) females, with an average age of 68.3 years (range, 42–91 years). All cases had positive results for SARS-CoV-2 by nucleic acid testing and characteristic radiologic alterations in lungs. Ten (41.7%) patients had a history of hypertension or coronary heart diseases or both. One patient had both coronary heart diseases and diabetes. Six (25%) patients had a history of tumor. Three patients had Schistosoma infection and one patient had obstructive cholestasis. One patient had a history of hepatocellular carcinoma. The clinical information and laboratory results are summarized in Tables 1, 2.

**Table 1:** Clinical information of patients with COVID-19 (*N* = 24).

**Demographics and combined diseases**		**Characters**
Age (years)		66 (60,78)
Sex (*n*, %)	Female	8 (33%)
	Male	16 (67%)
Chronic liver diseases (*n*, %)	With	4 (16.7%)
	Without	20 (83.3%)
Diabetes (*n*, %)	With	1 (4.2%)
	Without	23 (95.8%)
Hypertension (*n*, %)	With	8 (33.3%)
	Without	16 (66.7%)
Coronary heart disease (*n*, %)	With	5 (20.8%)
	Without	19 (79.2%)
Chronic kidney disease (*n*, %)	With	1 (4.2%)
	Without	23 (95.8%)
Cancer (*n*, %)	With	6 (25.0%)
	Without	18 (75.0%)

*All statistics are presented as median (interquartile range) or N (%)*.

**Table 2:** Summary of laboratory results.

	**Initial value**	**Peak value**
ALT (U/L) (*N* = 24)	30 (23.5, 34.75)	124 (46, 629.5)
AST (U/L) (*N* = 24)	42 (27.3, 58.5)	96 (60, 817)
LDH (U/L) (*N* = 24)	465 (246.8, 591)	614.5 (452.8, 2,072)
TBIL (umol/l) (*N* = 24)	12.2 (9.2, 21.3)	38.8 (20.3, 49.1)
ALP (U/L) (*N* = 24)	71.5 (60.8, 90.5)	111.5 (77, 178.8)
GGT (U/L) (*N* = 24)	40.5 (20.3, 65.8)	75.5 (38.8, 165.5)
Albumin (g/l) (*N* = 24)	27.8 (25.6, 31.8)	21.9 (17.3, 24.1)
hs-TnI (ng/l) (*N* = 18)	19.55 (7.7, 61.1)	227.2 (49.2, 1,876)
CK-MB (U/L) (*N* = 16)	14 (12, 22)	47 (26, 101.8)
WBC (G/L) (*N* = 24)	8.735 (5.5, 12.4)	21.5 (15.3, 27.8)
LY (G/L) (*N* = 24)	0.49 (0.33, 0.72)	0.24 (0.19, 0.31)
PLT (G/L) (*N* = 23)	146 (108, 233)	33 (22, 88)
Hb (g/l) (*N* = 23)	121 (105, 144)	70 (61, 87)
Cr (umol/l) (*N* = 24)	68.5 (61, 98.5)	122.5 (92.1, 208.4)
BUN (mmol/l) (*N* = 24)	7.2 (4.9, 11.2)	19.6 (15.8, 32.9)
D-dimer (ug/ml) (*N* = 24)	1.3 (0.7, 7.8)	8 (6.5, 8)
PCT (ng/ml) (*N* = 22)	0.14 (0.11, 0.37)	5.7 (2.3, 25.4)
CRP (mg/l) (*N* = 24)	82.8 (45.1, 118)	153.4 (118.4, 173.9)

*All statistics are presented as median (interquartile range)*.

*ALT, alanine aminotransferase; ALP, Alkaline phosphatase; AST, aspartate aminotransferase; BUN, blood urea nitrogen; CK, creatine kinase; Cr, creatinine; CRP, C-reactive protein; DM, diabetes; GGT, γ-glutamyl transpeptidase; Hb, hemoglobin; hs-TnI, high sensitive troponin I; LDH, lactate dehydrogenase; LY, lymphocytes; PCT, procalcitonin; PLT, platelet; TBIL, total bilirubin; WBC, white blood cell*.

### Light Microscopy Findings

Light microscopy showed that all liver sections had different degrees of liver injury manifested as swelling of the hepatocytes, hepatocellular necrosis, steatosis, lobular inflammation, portal inflammation, dilatation of sinusoids, cholestasis, and fibrosis (Table 3). A detailed assessment of each feature is provided below.

**Table 3:** The pathologic abnormalities of liver using light microscopy observations in 24 cases of deceased patients with COVID-19.

**ID**	**Swelling of the hepatocytes**	**Cholestasis**	**Hepatocellular necrosis**	**Steatosis**		**Lobular inflammation**	**Portal inflammation**	**Fibrosis**	**GS**	**Dilatation of sinusoids**
				**Microvesicular steatosis (Relative area)**	**Macrovesicular steatosis**					
SJ1	Moderate	Mild	Mild	60%	0	2	1	1	G2S1	Moderate
SJ2	Moderate	N	Mild	70%	0	2	0–1	0	G2S0	Mild
SJ3	Moderate	Moderate	Mild	0	0	2	2	1	G2S1	Mild
SJ4	Mild	N	Mild	0	0	2–3	0	1	G2-3S1	Moderate
SJ5	Mild	Mild	Severe	5%	0	2–3	1	1	G2-3S1	N
SJ6	Mild	N	Mild	50%	0	1	1	0	G1S0	Mild
SJ7	Mild	N	Mild	10%	0	1	1	0	G1S0	N
SJ8	Moderate	Mild	Severe	50%	0	3	2	0	G3S0	Mild
SJ9	Moderate	N	Mild	50%	0	2	1	0	G2S0	Mild
SJ10	Mild	Mild	Severe	20%	0	3	1	0	G3S0	Moderate
SJ11	Mild	N	Moderate	40%	0	2	1	0	G2S0	Moderate
SJ12	Moderate	N	Moderate	10%	0	2	1	0	G3S0	Moderate
SJ13	Mild	N	Severe	50%	0	2	1	0	G2S0	Moderate
SJ14	Mild	N	Mild	80%	10%	2	1	0	G2S0	N
SJ15	Mild	N	Moderate	30%	50%	2–3	2	3	G2-3S3	Moderate
SJ16	Moderate	Mild	Severe	60%	0	3	1	0	G3S0	Mild
SJ17	Mild	N	Mild	70–80%	20%	2	2	1	G2S1	N
SJ18	Mild	N	Severe	80%	0	3	1	0	G3S0	Mild
SJ19	Mild	N	Severe	20%	0	3	1	0	G3S0	Mild
SJ20	Moderate	N	Moderate	70%	0	3	1	0	G3S0	Mild
SJ21	Moderate	Mild	Severe	0	0	4	2	0	G4S0	Mild
SJ22	Moderate	Mild	Severe	0	0	3	2	1	G3S1	N
SJ23	Moderate	N	Severe	10%	60%	2–3	1–2	0	G2-3S0	N
SJ24	Mild	N	Moderate	80%	10%	2	1–2	1	G2S1	N

*ID, identification number; N, no*.

**Figure 1:**
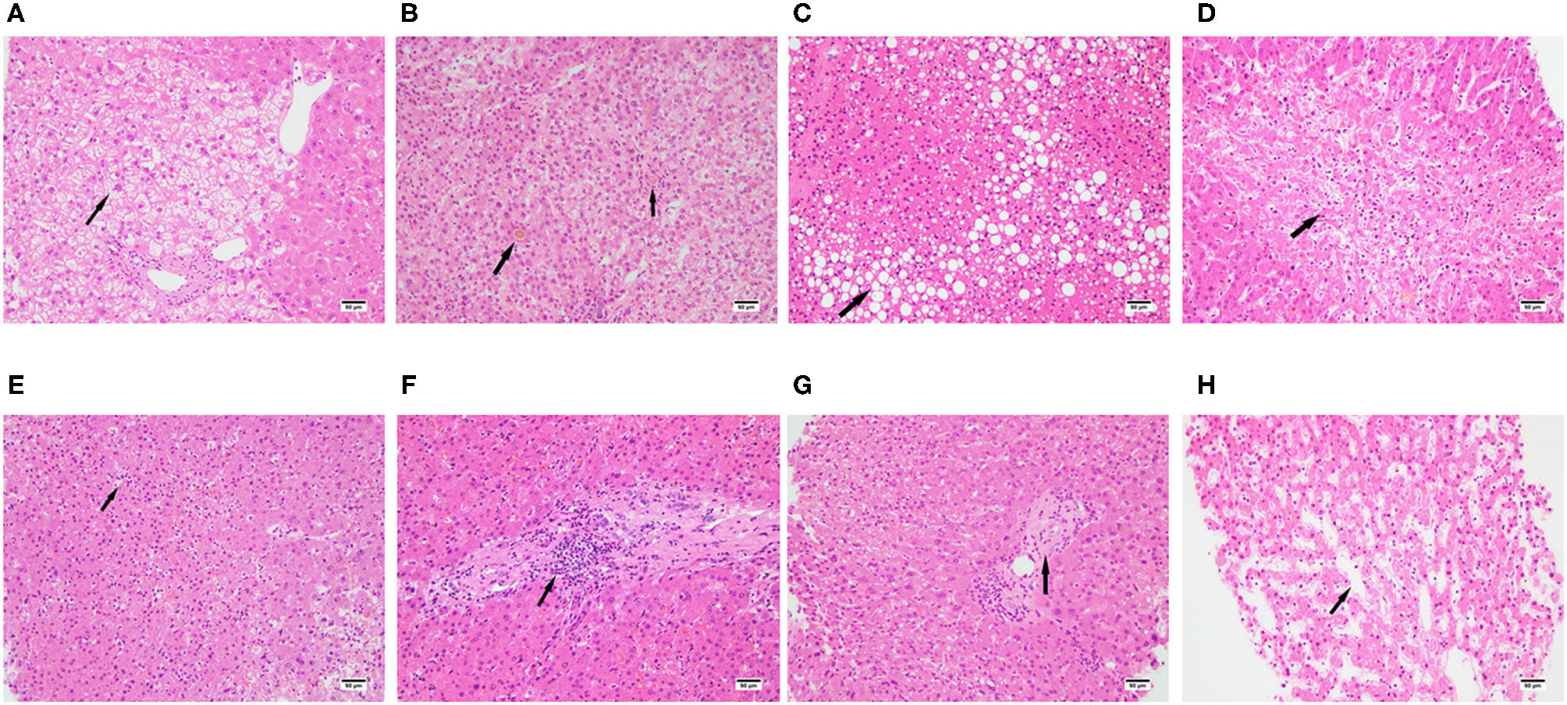
Spectrum of pathologic abnormalities of liver tissues from postmortem of patients with COVID-19. **(A)** swelling of the hepatocytes, **(B)** cholestasis, **(C)** steatosis, **(D)** hepatocellular necrosis. **(E)** lobular inflammation, **(F)** portal inflammation, **(G)** fibrosis, **(H)** dilatation of sinusoids, Bars = 50 μm **(A–D)**.

All cases presented with slight to moderate swelling of the hepatocytes (Figure 1A): 13 mild and 11 moderate. The degree of hepatocyte swelling was positively correlated with levels of LDH at both initial and peak time points, while there was no relationship between the swelling degree of the hepatocytes and therapeutic interventions (Supplementary Tables 1–3).

A previous study reported that about 38% of the patients dying of COVID-19 had cholestasis (14). Similarly, we found a total of eight from 24 patients (33.3%) had cholestasis (Figure 1B): 7 cases had mild cholestasis and one case had moderate cholestasis. There was no relationship between liver function tested at the initial time point and cholestasis. However, the levels of ALT, AST, and LDH tested at peak time point were positively correlated with cholestasis, and cholestasis was also correlated with antiviral therapy combined with ECMO intervention (Supplementary Tables 1–3).

Previous study found that 54–75% COVID-19 patients presented steatosis (11, 12, 14, 15). Consistently, we found 20 from 24 cases (83.3%) demonstrated steatosis, 15 cases presented microvesicular steatosis (Figure 1C) and the other 5 presented both microvesicular steatosis and Macrovesicular steatosis. There was no relationship between liver function test at both initial and peak time points and steatosis. Microvesicular steatosis was correlated with antiviral therapy combined with invasive mechanical ventilation intervention, antiviral therapy combined with ECMO intervention and invasive mechanical ventilation (Supplementary Tables 1–3).

28.6–47% of COVID-19 patients were reported to have hepatic necrosis (13, 15), while all cases with different degrees of hepatic necrosis were presented in our study, moderate necrosis was observed in 5 cases and severe hepatocellular necrosis was observed in 10 cases (Figure 1D). The level of total bilirubin at the initial time point was positively related to hepatic necrosis. The level of albumin at the peak time point was negatively related to hepatic necrosis. The levels of ALT, AST, and LDH at the peak time point were positively correlated with hepatic necrosis. Hepatic necrosis was also correlated with antiviral therapy combined with invasive mechanical ventilation intervention (Supplementary Tables 1–3).

Lobular inflammation was observed in all cases and most of them were moderate to severe (Figure 1E). The levels of ALT, AST, LDH, and PCT at the peak time point were positively correlated with lobular inflammation. Lobular inflammation was also correlated with antiviral therapy combined with ECMO intervention (Supplementary Tables 1–3).

Mild to moderate portal inflammation was observed in 23 cases (Figure 1F). The levels of ALT, AST, and LDH at the initial time point were positively correlated with portal inflammation while there was no relationship between portal inflammation and therapeutic intervention (Supplementary Tables 1–3).

Eight cases presented with hepatic fibrosis (Figure 1G). There was no relationship between liver function test at both initial and peak time points and hepatic fibrosis. There was also no relationship between hepatic fibrosis and therapeutic intervention (Supplementary Tables 1–3).

Mild to moderate dilatation of sinusoids were observed in 17 cases (Figure 1H). There was no relationship between liver function test at both initial and peak time points and dilatation of sinusoids. Dilatation of sinusoids was correlated with Umifenovir intervention (Supplementary Tables 1–3).

### Transmission Electron Microscopy Observations

**Figure 2:**
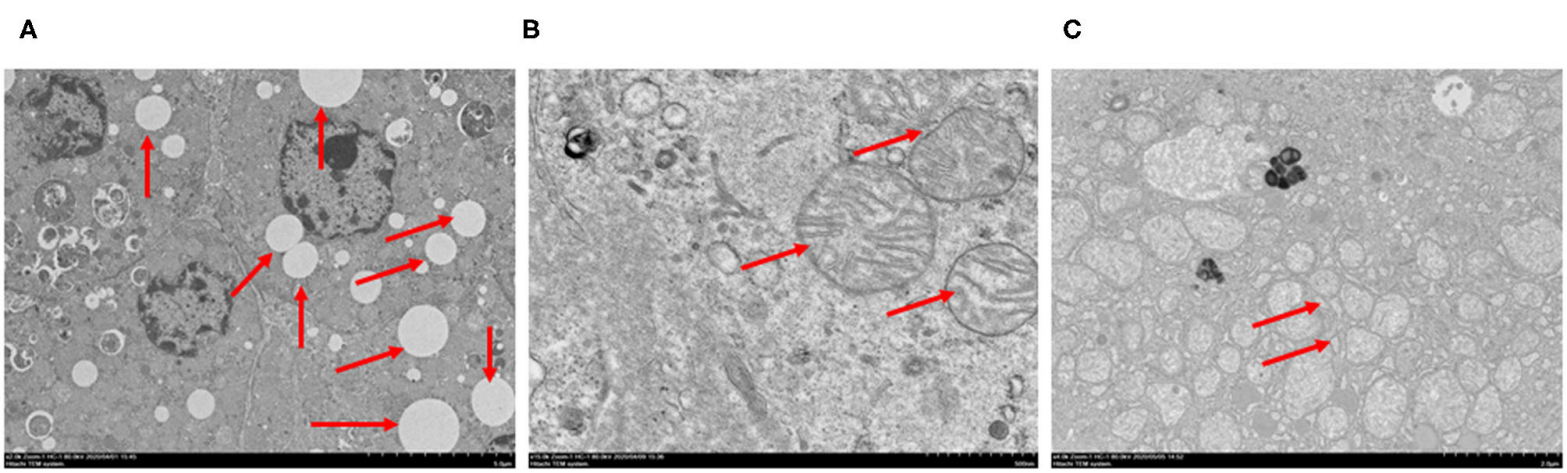
Spectrum of ultrastructural abnormalities of liver tissues from postmortem of patients with COVID-19. **(A)** vacuolar degenerations in hepatocytes (arrows), **(B)** edematous of mitochondria and the disruption of cristae (arrows), **(C)** expansion of endoplasmic reticulum (arrows). Bars = 5 μm **(A)**, 500 nm **(B)**, 2 μm **(C)**.

Coronavirus particles were also identified in the liver of deceased SARS-CoV-2 patients (16). In order to check whether there were viral particles or inclusions in our patients, we observed the liver tissues using transmission electron microscopy. Inconsistent with previous reports, coronavirus particles or viral inclusions were not detected in the liver tissues for any cases. Vacuolar degenerations in hepatocytes were noted (Figure 2A). Hepatocytes also exhibited marked edematous of mitochondria with the disruption of cristae (Figure 2B). The expansions of the endoplasmic reticulum were also observed (Figure 2C). These indicated that liver injury may be associated with other underlying conditions instead of the direct damage induced by Coronavirus.

### Liver Injury Is Not Associated With SARS-COV-2 Infection in Liver Tissue

Previous studies speculated that the virus may directly cause liver damage. In order to further confirm this hypothesis, we performed RT-PCR to detect SARS-COV-2 in liver tissues. Surprisingly, tests on the liver tissue from all cases were negative, which is consistent with the result from our transmission electron microscopy observations. This result indicated that liver injury in these cases is not directly caused by SARS-COV-2.

### The Degree of Liver Injury Is Associated With Severity of Hypoxemia in COVID-19

Twenty cases combined with respiratory failure. Among them, nine cases had severe hepatocellular necrosis and five cases had moderate hepatocellular necrosis, while severe hepatocellular necrosis was observed in one patient among the other four patients without respiratory failure. Severe hepatocellular necrosis and lobular inflammation were observed in two patients who were presented with severe respiratory failure and performed ECMO treatment. Moreover, microvesicular steatosis was correlated with invasive mechanical ventilation which is a therapeutic method for severe respiratory failure (Supplementary Table 3). These indicated that hypoxia may contribute to liver injury.

### The Degree of Liver Injury Is Not Associated With Combined Diseases in COVID-19

Three cases combined with Schistosoma infection presented with slight to moderate swelling of the hepatocytes, no or mild cholestasis, and mild to moderate hepatocellular necrosis. One case combined with obstructive cholestasis presented with moderate swelling of the hepatocytes, moderate cholestasis, and mild hepatocellular necrosis (Table 4). These results indicated that SARS-CoV-2 induced liver injury might be independent of pre-existing Schistosoma infection or obstructive cholestasis. Moreover, we analyzed the relationships between combined diseases and liver injury and found that there was no relationship between them (Supplementary Table 4).

**Table 4:** Exposure to hepatotoxic factors and treatment history.

**ID**	**Exposure to hepatotoxic factors**	**Antivirals**	**Antifungal drugs**	**Steroid**	**Invasive mechanical ventilation**	**CRRT**	**ECMO**
SJ1	Schistosoma infection	Umifenovir	Y	Y	Y	N	N
SJ2	N	Umifenovir, Ribavirin	N	Y	Y	Y	N
SJ3	Obstructive cholestasis	Umifenovir, Ribavirin	N	Y	N	N	N
SJ4	Schistosoma infection	Umifenovir, Ribavirin	N	N	N	N	N
SJ5	N	Umifenovir	Y	Y	Y	N	N
SJ6	N	Umifenovir, Ribavirin	N	Y	Y	Y	N
SJ7	N	Umifenovir	N	N	N	N	N
SJ8	N	Umifenovir, Ribavirin	Y	Y	Y	N	N
SJ9	N	Umifenovir	Y	Y	Y	Y	N
SJ10	N	N	N	Y	N	N	N
SJ11	N	Ganciclovir, Oseltamivir, Umifenovir	N	Y	Y	N	N
SJ12	N	Umifenovir, Interferon α2β	Y	Y	Y	N	N
SJ13	N	Oseltamivir, Umifenovir, Ribavirin, Interferon α2β, Lopinavir and Ritonavir	Y	Y	Y	N	N
SJ14	N	Interferon α2β, Ganciclovir, Ribavirin	N	Y	Y	N	N
SJ15	N	Umifenovir, Ribavirin	Y	Y	Y	N	N
SJ16	N	Umifenovir, Ribavirin	Y	Y	Y	N	N
SJ17	N	Umifenovir	Y	Y	Y	Y	N
SJ18	N	Umifenovir, Ribavirin	Y	Y	Y	Y	N
SJ19	N	Umifenovir, Ribavirin	Y	Y	Y	N	N
SJ20	N	Umifenovir	Y	N	Y	Y	N
SJ21	N	Umifenovir, Ribavirin, Interferon α2β	Y	Y	Y	Y	Y
SJ22	N	Ribavirin	Y	Y	Y	Y	Y
SJ23	N	Interferon α2β, Umifenovir, Ribavirin	Y	Y	Y	N	N
SJ24	Schistosoma infection	Ribavirin, Ganciclovir	N	Y	Y	Y	N

*CRRT, continuous renal replacement therapy; ECMO, Extracorporeal Membrane Oxygenation; ID, identification number; N, no; Y, yes*.

## Discussion

In the present study, we report the liver histopathologic, ultrastructural, and RT-PCR findings from biopsies of 24 patients who died from respiratory failure or circulatory failure due to COVID-19. This is the first detailed report of liver pathologic presentations using both light microscopy and transmission electron microscopy observations from a large series of liver tissues in patients with SARS-CoV-2 infection in China. Our biopsy study demonstrates the range of abnormalities present and the specific hepatocellular necrosis that might be induced by hypoxia in COVID-19 patients, and thus may provide important information for future clinicopathologic studies in severely ill patients with COVID-19 infection and liver injury. We observed mild to moderate cholestasis. This is in accordance with mechanisms known for SARS-CoV-2 infection via angiotensin-converting enzyme II (ACE2) on bile duct epithelial cells in the liver (20). We also show findings that suggest distinct mechanisms of this novel coronavirus infection, significant hepatocellular necrosis. Thus, these pathologic observations may provide a basis for further understanding of COVID-19.

The detailed description of pathologic findings has been described by several studies. Hepatic necrosis, portal tract inflammation, lobular inflammation, steatosis, increased number of histiocytes and megakaryocytes and platelet-fibrin microthrombi were described by Zhao et al. (15). Bradley et al. demonstrated the presence of acute congestion, centrilobular necrosis and mild periportal lymphocytic inflammation (13). Sonzogni et al. reported fibrosis, mild to moderate lobular inflammation, mild portal inflammation, parenchymal confluent necrosis, steatosis, vascular thrombosis, vascular alterations including portal vein parietal fibrosis, herniated portal vein in periportal parenchyma and periportal abnormal vessels (11). Lagana et al. found lobular necroinflammation and portal inflammation, lobular apoptosis, steatosis, and cholestasis (14). Diaz et al. wrote a meta-analysis to summarize the presentations including hepatic steatosis, congestion of hepatic sinuses and necrosis, vascular thrombosis and other vascular alterations, fibrosis, Kupffer cell proliferation or hyperplasia, portal and lobular inflammation (12). We found similar presentations including hepatocellular necrosis, cholestasis, steatosis, lobular inflammation, portal inflammation and fibrosis. Moreover, we found swelling of the hepatocytes, and dilatation of sinusoids.

Vascular thrombosis was described in several articles (11, 12, 15), while we did not find vascular thrombosis in our paper. This may be related with a lower level of platelets and D-dimer value compared to previous study. Only five patients had a low level of platelets (<100 G/L) in the study of Sonzogni et al. (11), while a total of 19/23 (82.6%) patients had a low level of platelets (<100 G/L) with an average of 33 G/L at peak time point in our paper. The average value of D-dimer was 8 ug/ml (80 ng/dl) in our paper, which is lower than previous report with 96% patients having a very high value of D-dimer values ≥500 ng/dL (11).

In this study, we did not detect viral RNA or viral particles from liver tissues for all patients. Lagana et al. identified viral RNA in some liver tissue samples, but there was no significant correlations between PCR positivity and any histologic finding (14). Thus, we conclude that liver damage was not caused by virus directly. We observed significant hepatocellular necrosis, and the change of ultrastructural features of hepatocytes, such as vacuolar degenerations, edematous of mitochondria with the disruption of cristae and the expansions of endoplasmic reticulum in cases without viral particles or inclusions detected in their liver tissues. These indicate that hepatocellular injury may be induced by other mechanisms instead of SARS-CoV-2 infection directly. Hypoxia, presenting as one of the common symptoms in COVID-19 patients, may be related to liver injury (21). Our data confirmed that more patients with severe hepatocellular necrosis had respiratory failure compared to patients with mild or moderate hepatocellular necrosis. Moreover, microvesicular steatosis was correlated with invasive mechanical ventilation which is a therapeutic method for severe respiratory failure. We also found that the level of PCT at the peak time point was positively correlated with lobular inflammation. Thus, systemic inflammation induced by virus and hypoxia which is induced by respiratory failure might contribute to liver injury. Moreover, organ failure may be also related with liver injury, as invasive mechanical ventilation and/or ECMO intervention which were therapeutic methods for organ failure were related with liver injury. Besides, the adverse effects of some drugs may be another factor to cause liver injury (22, 23). In our study, antiviral drugs were widely used for our patients, and patients treated with two or more kinds of antiviral drugs seem to have more severe hepatocyte necrosis and liver injury (Table 3). In addition, patients who had a history of antifungal drugs usage seem to have more severe hepatocyte necrosis and liver injury (Table 3).

Our data confirmed that 41.7% of the patients with liver injury presented as severe hepatocellular necrosis, which was higher than those found in previous studies (24, 25). This might be related to the severity of the COVID-19 patients. About 80% of the patients were non-severe patients in previous studies (24, 25), while all the patients in our study were severe cases and deceased in the end. Besides, the mechanisms of liver injury might be different with different degrees of severity of COVID-19 infection.

In this study, we did not detect viral RNA, viral particles, or inclusions from liver tissues for all patients, which is inconsistent with previous studies (11, 14–16). It is probable that there was no virus in the liver tissue for these patients. It is also possible that the virus was cleared already, and the clearance of the virus may be related to different stages of disease progression.

In summary, we observed pathologic changes of liver injury using light microscopy and transmission electron microscopy in some deceased patients with COVID-19. We described the liver injury patterns and the severity of liver injury and found that 41.7% of the patients had severe hepatocyte necrosis. Liver injury may be related with systemic inflammation and hypoxia induced by COVID-19 infection, hepatotoxic effects from antifungal agents and antiviral therapy, and organ failure that needs invasive mechanical ventilation and/or ECMO intervention. However, there are still some limitations for our study, such as a relatively small number of cases. Further studies with a larger number of cases are needed for more comprehensive understandings of liver injury in COVID-19 patients.

## Data Availability Statement

The original contributions presented in the study are included in the article/Supplementary Material, further inquiries can be directed to the corresponding author/s.

## Ethics Statement

The studies involving human participants were reviewed and approved by the Institutional Ethics Board of Union Hospital, Tongji Medical College, Huazhong University of Science and Technology. The next of kin of each enrolled patient/participant provided their written informed consent to participate in this study. Written informed consent was obtained from the individual(s) for the publication of any potentially identifiable images or data included in this article.

## Author Contributions

HC: conceptualization, methodology, validation, formal analysis, investigation, and writing—original draft. LP: methodology, validation, formal analysis, investigation, and visualization. LH and JZ: methodology, validation, and formal analysis. YZ: methodology, formal analysis, and language correction. HS: formal analysis, investigation, and visualization. LYao and QZ: methodology, validation, formal analysis, and investigation. XN, LYan, and XH: conceptualization, investigation, resources, writing—review and editing, and supervision. All authors contributed to the article and approved the submitted version.

## Funding

The study was supported by Urgent projects of scientific and technological research on COVID-19 funded by Hubei province (No. 2020FCA014 to XH), Key Special Projects of the Hubei Provincial Department of Science and Technology (No. 2019ACA133 to LY), National Natural Science Foundation of China (No. 82072333 to XN), and the China Postdoctoral Science Foundation (No. 2020M670289 and 2020T130065ZX to JZ).

## Conflict of Interest

The authors declare that the research was conducted in the absence of any commercial or financial relationships that could be construed as a potential conflict of interest.

## Publisher's Note

All claims expressed in this article are solely those of the authors and do not necessarily represent those of their affiliated organizations, or those of the publisher, the editors and the reviewers. Any product that may be evaluated in this article, or claim that may be made by its manufacturer, is not guaranteed or endorsed by the publisher.
