# Supplementary material for: Liver Histopathological Analysis of 24 Postmortem Findings of Patients With COVID-19 in China
Source: Front Med (Lausanne). 2021 Oct 11;8:749318. doi: 10.3389/fmed.2021.749318 (PMC8543004; doi:10.3389/fmed.2021.749318)
Supplement: Supplementary file 1 [file Data_Sheet_1.PDF]

Supplementary files

Liver histopathological analysis of 24 postmortem findings of patients with COVID-19 in China

Huikuan Chu[#1], Li Peng[#2], Lilin Hu[1], Yixin Zhu[3], Jinfang Zhao[4], Hua Su[5], Lin Yao[1], Qingjing Zhu[6], Xiu Nie[2*], Ling Yang[*1], Xiaohua Hou[*1]

[1]Division of Gastroenterology, Union Hospital, Tongji Medical College, Huazhong University of Science and Technology, 1277 Jiefang Avenue, Wuhan 430022, China

[2]Department of Pathology, Union Hospital, Tongji Medical College, Huazhong University of Science and Technology, 1277 Jiefang Avenue, Wuhan 430022, China

[3]Department of Medicine, University of California San Diego, La Jolla, CA, USA

[4]Center for Life Sciences, Tsinghua University, Beijing, China

[5]Department of Nephrology, Union Hospital, Tongji Medical College, Huazhong University of Science and Technology, 1277 Jiefang Avenue, Wuhan 430022, China

[6]Liver and Infectious Diseases Department, Wuhan Jinyintan Hospital, Wuhan, China, Dongxihu District, Wuhan 430023

[#] These authors contributed equally to this paper.

* These authors shared co-coresponding authors.

*** Correspondence**

Xiu Nie,M.D., Department of Pathology, Union Hospital, Tongji Medical College, Huazhong University of Science and Technology, 1277 Jiefang Avenue, Wuhan 430022, China

Ling Yang, M.D., Ph.D. Division of Gastroenterology, Union Hospital, Tongji Medical College, Huazhong University of Science and Technology, 1277 Jiefang Avenue, Wuhan, 430022, China.

Email: hepayang@163.com;

phone +86-2785726678, +8613971178791

Xiaohua Hou, M.D., Ph.D. Division of Gastroenterology, Union Hospital, Tongji Medical College, Huazhong University of Science and Technology, 1277 Jiefang Avenue, Wuhan, 430022, China.

Email: houxh@hust.edu.cn
Phone +86 -2785726678, +8613035143646

Supplementary Table 1 The relationships of pathologic changes and clinical liver function and inflammatory data tested at initial time point

Initial value		Swelling of the hepatocytes	Cholestasis	Hepatocellular necrosis	Steatosis		Lobular inflammation	Portal inflammation	Fibrosis	Dilatation of sinusoids
					Microvesicular steatosis	Macrovesicular steatosis				
Albumin (g/l)	rho	-0.344	-0.224	-0.126	-0.024	0.251	-0.004	0.146	0.343	-0.046
	P	0.108	0.304	0.567	0.916	0.249	0.986	0.505	0.109	0.834
ALT (U/L)	rho	0.139	0.173	0.213	-0.112	0.134	0.069	0.43	-0.025	-0.107
	P	0.516	0.419	0.317	0.611	0.533	0.75	*	0.908	0.619
AST (U/L)	rho	0.327	0.267	-0.128	-0.26	-0.025	0.013	0.553	0.161	-0.261
	P	0.119	0.207	0.55	0.232	0.906	0.952	**	0.452	0.219
LDH (U/L)	rho	0.526	0.294	0.32	-0.307	-0.033	0.203	0.524	0.078	0.02
	P	**	0.163	0.128	0.155	0.877	0.342	**	0.716	0.927
TBIL (umol/l)	rho	0.053	-0.175	0.46	-0.024	0.094	0.279	0.279	-0.149	-0.042
	P	0.81	0.426	*	0.916	0.671	0.197	0.198	0.498	0.849
ALP (U/L)	rho	0.024	0	-0.132	-0.076	0.078	-0.206	-0.073	-0.123	-0.02
	P	0.911	1	0.54	0.73	0.716	0.335	0.734	0.567	0.927
GGT (U/L)	rho	0.175	-0.103	-0.244	-0.016	0.133	-0.178	0.009	-0.209	0.233
	P	0.413	0.632	0.251	0.943	0.536	0.404	0.966	0.327	0.274
PCT (ng/ml)	rho	0.277	0.362	-0.153	-0.362	-0.428	-0.242	-0.234	0.18	0.417
	P	0.36	0.224	0.617	0.224	0.144	0.427	0.442	0.557	0.156
CRP (mg/l)	rho	0.197	-0.167	-0.071	0.281	-0.16	-156	-0.299	-0.293	0.317
	P	0.368	0.446	0.747	0.206	0.941	0.477	0.166	0.175	0.14

Data are given as Spearman's rho correlation coefficients and P-value. **P < 0.05*, ***P < 0.01.*

ALT, alanine aminotransferase; ALP, Alkaline phosphatase; AST, aspartate aminotransferase; CRP, C-reactive protein; GGT, γ-glutamyl transpeptidase; LDH, lactate dehydrogenase; PCT, procalcitonin; TBIL, total bilirubin,

Supplementary Table 2 The relationships of pathologic changes and clinical liver function and inflammatory data tested at peak time point

Peak value		Swelling of the hepatocytes	Cholestasis	Hepatocellular necrosis	Steatosis		Lobular inflammation	Portal inflammation	Fibrosis	Dilatation of sinusoids
					Microvesicular steatosis	Macrovesicular steatosis				
Albumin (g/l)	rho	0.198	-0.02	-0.459	0.24	0.051	-0.201	-0.095	0.233	0.067
	P	0.364	0.929	*	0.282	0.818	0.358	0.667	0.284	0.761
ALT (U/L)	rho	0.272	0.491	0.793	-0.259	-0.307	0.574	0.272	-0.318	0.126
	P	0.199	*	***	0.232	0.144	**	0.199	0.13	0.557
AST (U/L)	rho	0.338	0.617	0.725	-0.235	-0.334	0.587	0.283	-0.262	0.051
	P	0.106	**	***	0.281	0.11	**	0.181	0.216	0.812
LDH (U/L)	rho	0.405	0.585	0.666	-0.256	-0.173	0.551	0.336	-0.107	0.134
	P	*	**	***	0.239	0.42	**	0.109	0.619	0.533
TBIL (umol/l)	rho	0.066	0.111	0.341	-0.241	0.058	0.343	0.308	0.095	-0.328
	P	0.764	0.615	0.112	0.281	0.794	0.109	0.153	0.667	0.127
ALP (U/L)	rho	0.145	0.323	0.067	-0.221	-0.154	-0.05	0.108	0.018	0.071
	P	0.499	0.123	0.756	0.311	0.473	0.816	0.614	0.932	0.742
GGT (U/L)	rho	0.211	0.056	-0.111	-0.074	0.14	-0.154	0.284	-0.026	0.102
	P	0.321	0.793	0.605	0.737	0.515	0.474	0.178	0.903	0.634
PCT (ng/ml)	rho	0.233	0.393	0.27	0.128	-0.192	0.424	0.253	-0.005	0.152
	P	0.297	0.07	0.224	0.579	0.391	*	0.256	0.983	0.499
CRP (mg/l)	rho	0.054	-0.81	0.329	0.157	0.262	0.155	0.33	-0.041	0.126
	P	0.801	0.708	0.117	0.473	0.217	0.47	0.115	0.849	0.557

Data are given as Spearman's rho correlation coefficients and P-value. **P < 0.05*, ***P < 0.01*, ****P < 0.001.*

ALT, alanine aminotransferase; ALP, Alkaline phosphatase; AST, aspartate aminotransferase; CRP, C-reactive protein; GGT, γ-glutamyl transpeptidase; LDH, lactate dehydrogenase; PCT, procalcitonin; TBIL, total bilirubin,

Supplementary Table 3 The correlations of pathologic changes and therapeutic intervention

		Umifenovir	Ribavirin	Ganciclovir	Interferon α2β	Combination of antiviral drugs	Antiviral drugs combined with steroid	Antiviral drugs combined with invasive mechanical ventilation	Antiviral drugs combined with CRRT	Antiviral drugs combined with ECMO	Invasive mechanical ventilation
Swelling of the hepatocytes	U	25	58	13.5	38	48.5	25	25	55	9	30
	P	0.598	0.881	0.082	0.547	0.563	0.598	0.598	0.561	0.131	0.37
Cholestasis	U	29.5	54	19.5	38.5	47.5	19.5	26	53.5	6	29
	P	0.955	0.63	0.233	0.547	0.48	0.233	0.65	0.457	*	0.301
Hepatocellular necrosis	U	30	39	23	31	42	16	9	56	7	24.5
	P	1	0.146	0.494	0.264	0.316	0.171	*	0.637	0.102	0.197
Microvesicular steatosis	U	22	54	11	38.5	55	25.5	8	34.5	3	13
	P	0.45	0.689	0.073	0.616	0.945	0.671	*	0.063	*	*
Macrovesicular steatosis	Chi-square value	-	-	-	-	-	-	-	-	-	-
	P	0.107	0.621	0.107	0.291	1	1	1	1	1	0.544
Lobular inflammation	U	27	42.5	22.5	43	51.5	25	14.5	51.5	5	31
	P	0.751	0.191	0.428	0.863	0.728	0.597	0.102	0.402	*	0.425
Portal inflammation	U	18	46.5	28.5	39	56	13.5	24	55.5	6	31
	P	0.214	0.323	0.877	0.612	1	0.088	0.535	0.592	0.064	0.425
Fibrosis	Chi-square value	-	-	-	-	-	-	-	-	-	-
	P	0.269	1	1	0.621	0.182	1	0.269	1	1	0.578
Dilatation of sinusoids	U	6	57.5	22.5	38	33.5	29	29	42	13.5	31.5
	P	*	0.863	0.464	0.577	0.108	0.922	0.922	0.157	0.381	0.483

**P* < 0.05.*

Supplementary Table 4 The correlations of pathologic changes and combined diseases

		HT	DM	CHD	Cancer	History of HT, CHD, DM, CKD or cancer
Swelling of the hepatocytes	U	60	6	32	45	57
	P	0.777	0.358	0.202	0.487	0.469
Cholestasis	U	55	7.5	39	39.5	54.5
	P	0.504	0.483	0.464	0.241	0.347
Hepatocellular necrosis	U	59.5	4	38.5	52.5	63
	P	0.767	0.245	0.492	0.914	0.773
Microvesicular steatosis(Relative area)	U	60	7.5	35	45	50.5
	P	0.8	0.55	0.359	0.535	0.295
Macrovesicular steatosis	Chi-square value	-	-	-	-	-
	P	0.631	1	1	1	0.326
Lobular inflammation	U	44	0.5	46.5	50	62.5
	P	0.161	0.069	0.935	0.76	0.733
Portal inflammation	U	46	8.5	35.5	50	50.5
	P	0.208	0.62	0.33	0.76	0.247
Fibrosis	Chi-square value	-	-	-	-	-
	P	0.667	1	1	1	0.412
Dilatation of sinusoids	U	38.5	3	30.5	37	59
	P	0.096	0.19	0.198	0.227	0.589

CHD, coronary heart disease; CKD, chronic kidney disease; DM, diabetes; HT, hypertension.
